# Supplementary material for: Learning curve and surgical outcome of robotic assisted colorectal surgery with ERAS program
Source: Sci Rep. 2022 Nov 29;12:20566. doi: 10.1038/s41598-022-24665-w (PMC9709162; doi:10.1038/s41598-022-24665-w)
Supplement: Supplementary file 2 — Supplementary Information 2. [file 41598_2022_24665_MOESM2_ESM.zip › RAL Raw data-Ñ[▒K/060/OP N_ñw╝╨░O▒Kñσ.pdf]

W52 / W52

臺 中 榮 民 總 醫 院
手術室護理記錄

[redacted]

基本資料

- 室別： 開刀房-17室，手術科別： CRS，手術類別： 常規，日期： 2021/02/05
- 進入時間： 0905 ； 麻醉開始時間： 0910 ； Time Out時間： 0940 ； 劃刀時間： 0941 ； 手術結束時間： 1400 送出時間： 1510 ；
- 麻醉方式： GE

  診斷： Malignant tumor of rectum,L/3

  手術名稱： Robot Assisted Low Anterior Resection

  手術局部用藥： NIL

  術前預防性抗生素：
    - 抗生素名稱一： Veterin 1GM IVD由麻醉人員依醫囑給予
    - 時間： 0940
    - 上次簽章: 廖子晴

  ，追加預防性抗生素：
    - 抗生素名稱一： Veterin 1GM IVD由麻醉人員依醫囑給予
    - 時間： 1340
    - 上次簽章: 紀玉娟
- 手術醫師： 蔣鋒帆
- 麻醉醫師： VS 張詒婷
-
    - 刷手護士A ： 紀玉娟，時間： 0905，刷手護士B ： 鄭鈞羅，時間： 0905，接替刷手一： 鄭鈞羅/陳心慈，時間： 1200，接替刷手二： 廖子晴，時間： 1500
    - 流動護士A ： 廖子晴，時間： 0905，接替流動一： 紀玉娟，時間： 1230，接替流動二： 陳心慈，時間： 1500

皮膚狀況

術前： 完整

記錄者： 上次簽章: 廖子晴

術後： 完整

記錄者： 陳心慈

手術準備

手術臥式 單一手術臥式

-
    - 手術臥式： 截石臥式
    - 使用附件： 溫毯 38 ℃，頭圈，腳架
    - 皮膚準備： 10%Aq Betadine，Hibiscrub，2% Chlorhexidine
    - 電燒： 使用電燒: 電極片位置： 右大腿，記錄者： 上次簽章: 廖子晴

止血帶T： 無

植入物

- 植入物名稱/號碼/數量/部位： da vinci xi endowrist stapler 45mm*4.3mm*2
  covidien GIA80mm*3.8mm*1
  ethicon endo-surgery curved intraluminal stapler 29mm*1/rectum

，記錄者： 上次簽章: 紀玉娟

標本送檢

- 細菌培養 無
- 細胞學 無
- 冰凍切片 無
- 病理標本

  組織名稱一：rectum

  ，

組織名稱二： IMA root

,

組織名稱三： distal cut end

- 截肢 無

標本交付 標本： 1 袋 ，標本： 0 盒 ，標本： 2 瓶 ，細胞學： 0 管 ，Culture Tube： 0 管 ，被交付醫師： CR 楊曙榮，

- 記錄者： 上次簽章: 紀玉娟
- 時間： 1455

傷口術後敷料

傷口敷料： 傷口敷料： 無

- 請於圖上標示部位：

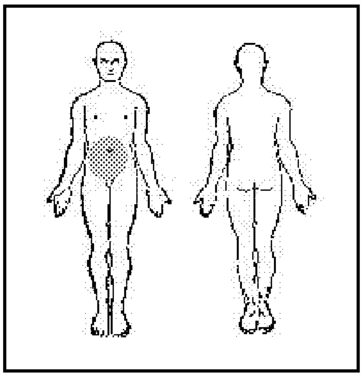

- 傷口敷料： 軟性敷料

導管

導管	天數	導管資訊	插入日期	插入時間	更換日期	拔除日期	拔除時間	拔除原因	預計拔除/更換	預計拔除/更換日期
A-line (1)	1	號碼:20 Ga; 部位:左手;	2021/02/05	0935						
Epidural PCA(1)	1	部位:脊髓;	2021/02/05	0830						
Double Lumen CVC(1)	1	號碼:8 Fr; 深度:15cm; 部位:jugular (右); 象限:RU;	2021/02/05	0937						
Foley(1)	1	號碼:16 Fr.; 部位:膀胱; 註記:balloon10c.c;	2021/02/05	0920						

用物計數

- 手術中出血量： 少 c.c.
- 手術中尿量： 400 c.c.

用物計數： 多切口同時縫合

- 1.器械： 正確 ，2.縫針： 正確 ，3.刀片： 正確 ，4.紗布： 正確 ，5.腹部墊： 未使用 ，6.紗球： 未使用 ，7.PEANUTS ： 未使用 ，8.紗條： 未使用 ，9.絲帶： 未使用 ，10.針頭： 未使用 ，11.Vessel loop： 未使用 ，12.Boots： 未使用 ，13.Jelco： 未使用 ，14.棉條： 未使用 ，
  - 第一次：
    - 刷手護士 ： 鄭鈞躍
    - 流動護士： 上次簽章: 紀玉娟
    - 時間： 1415

,

  - 第二次：
    - 刷手護士 ： 鄭鈞躍
    - 流動護士： 上次簽章: 紀玉娟
    - 時間： 1425

,

  - 第三次：
    - 刷手護士 ： 鄭鈞躍
    - 流動護士： 上次簽章: 紀玉娟
    - 時間： 1435

備註

備註： Davinci "XI"scope No.2
腹腔鏡NO.1

- 記錄者： 上次簽章: 廖子晴
- 時間： 0940

填寫護理人員 陳心慈

正式摘要

W52 / W52

doctorRank:B
